# Delivery Systems to Enhance Bioaccessibility and Bioavailability of Bioactive Factors: Structure, Property, and Food Applications

**DOI:** 10.3390/foods12163127

**Published:** 2023-08-20

**Authors:** Yaqiong Zhang, Xin Jia

**Affiliations:** 1 School of Agriculture & Biology, Shanghai Jiao Tong University, Shanghai 200240, China; 2 College of Food Science & Nutritional Engineering, China Agricultural University, Beijing 100083, China; xinjia@cau.edu.cn

Incorporating bioactive factors to strengthen food nutrition is important for functional food development. However, these factors are usually easily degraded in the presence of light, heat, oxygen, and the gastrointestinal environment, resulting in reduced bioaccessibility and bioavailability, which greatly limits their application in food processing. In recent years, there has been an emerging interest in the development of well-designed delivery systems to encapsulate and protect bioactive factors before they are introduced into the final product, which can preserve the quality of bioactive factors and enhance their applicability to food formulations. Many of these delivery systems are particles with nano- or micro-sizes, or three-dimensional cross-linked gel networks made of biopolymers that are able to retain a significant quantity of water without dissolving in it. The tunable nature of the structures in delivery systems makes it possible to enhance their physicochemical and functional properties via chemical or physical modifications, enabling various applications. Meanwhile, properties such as low toxicity, biodegradability, and environmental responsiveness make them more advantageous in the applications of nutrient and drug delivery systems.

This Special Issue aims to highlight encapsulation-based delivery systems to enhance the bioaccessibility and bioavailability of bioactive factors, with a particular attention to the development of new biopolymer-based encapsulation materials using chemical or enzymatic modification methods to fabricate delivery systems (particles, hydrogels, emulsion gels, etc.). It also includes in-depth studies on carrier structural design, dynamic structure–activity relationships between carrier structures and the controlled release of bioactive factors during digestion to provide insights into factors affecting the bioaccessibility and bioavailability of bioactive factors. As a result, this Special Issue includes five valuable scientific contributions. 

A novel microbial transglutaminase-induced cross-linked sodium caseinate (MSC) was synthesized to stabilize zein nanoparticles. These zein-MSC nanoparticles, which showed the desired stability, were used as an encapsulation carrier for resveratrol. Compared with free resveratrol, the photo-stability and bioaccessibility of resveratrol-loaded zein-MSC nanoparticles were significantly improved. The cellular studies also showed that resveratrol-loaded zein-MSC nanoparticles exhibited lower cytotoxicity and desirable anti-inflammatory activity [1].

Li et al. fabricated a type of oral starchy colon-targeting delivery system, which could be used to improve the bioaccessibility of bioactive insulin. By using layer-by-layer assembly of starchy polyelectrolytes (carboxymethyl anionic starch and spermine cationic starch) onto the surface of insulin nanoparticles via electrostatic interaction, insulin nanoparticles could be protected from degradation by digestive fluids, thus avoiding their burst release in simulated gastric and intestinal fluids [2].

Liao et al. developed alginate-based multilayered gel microspheres for the layered encapsulation and simultaneous delivery of vitamin B2 and β-carotene. It was found that the alginate concentration and the number of layers had notable effects on the mechanical properties and particle size of the gel microspheres. Meanwhile, the multilayered gel structure possessed the characteristics of pH response and excellent thermal stability, which could protect vitamin B2 and β-carotene from the intestinal environment, and further, markedly improve their bioaccessibility and bioavailability [3].

Dual-induced soy protein isolate-sugar beet pectin emulsion gel was fabricated as a novel oral delivery carrier to co-load hydrophilic riboflavin and lipophilic β-carotene simultaneously. It was found that the induction method could influence the structure and digestion pattern of emulsion gels, which further achieved controlled release of the encapsulated bioactive factors in simulated digestive fluids [4].

Chitosan, sodium alginate, and sodium tripolyphosphate were used as materials for the preparation of composite hydrogels, which exhibited excellent pH sensitivity and Ganoderma lucidum peptides (GLP) loading capacity. After encapsulation, the digestion and thermal stability of GLP was improved, while the antioxidant activity of GLP was retained. This study suggested that the composite hydrogels had a great deal of potential as a peptide carrier for oral delivery [5].

In summary, the Special Issue “Delivery Systems to Enhance Bioaccessibility and Bioavailability of Bioactive Factors: Structure, Property, and Food Applications” shows some of the latest advances in the development and design of novel carrier materials and structures for encapsulating bioactive factors, which could improve the physicochemical and functional properties of bioactive factors and enhance their applicability in food formulations. Moreover, the potential biological fate is one of the most important functional characteristics of delivery systems. Although great developments of analytical approaches for characterizing the biological fate of delivery systems have been achieved, most studies are limited to assessing the bioaccessibility of bioactive factors using different in vitro static and dynamic models that simulate the digestive conditions of the GIT. However, the key stage of oral bioavailability is the passage of bioactive factors from the intestinal lumen into the epithelial cells, and then into the systemic circulation. Therefore, more approaches using cell culture models, animal studies, and clinical studies are needed in the future to confirm the oral bioavailability of bioactive factors after being encapsulated into different delivery systems. 
